# La broncholithiase: une complication rare de la tuberculose pulmonaire

**DOI:** 10.11604/pamj.2015.20.206.6362

**Published:** 2015-03-06

**Authors:** Nabil Hammoune, Hicham Janah

**Affiliations:** 1 Service de Radiologie, Troisième Hôpital Militaire, Laayoune, Maroc; 2 Service de Pneumologie, Troisième Hôpital Militaire, Laayoune, Maroc

**Keywords:** Broncholithiase, hémoptysie, tuberculose pulmonaire, Broncholithiasis, hemoptysia, pulmonary tuberculosis

## Image en medicine

La broncholithiase est une affection rare, caractérisée par la présence de concrétions calcaires dans la lumière bronchique. Nous rapportons un cas de broncholithiase révélé par des crachats hémoptoiques, ayant comme antécédent une tuberculose pulmonaire étendue. La radiographie du thorax a montré des opacités intraparenchymateuses de tonalité calcique. La TDM thoracique a révélé des dilatations des bronches cylindriques, un épaississement peribronchovasculaire avec multiples calcifications intra-parenchymateuses évoquant des broncholithiases sur lésions séquellaires d'origine tuberculeuse. L'endoscopie bronchique a montré un aspect inflammatoire avec image de broncholite. L'abstention thérapeutique avec une surveillance a été décidée. La broncholithiase est une pathologie rare mais potentiellement grave. Ses manifestations cliniques ne sont pas spécifiques puisque la lithoptysie qui est pathognomonique de cette maladie est rare. Les principales causes sont la tuberculose, l'histoplasmose, et la silicose. Le diagnostic de certitude repose sur la combinaison de la TDM et de l'endoscopie. Le traitement s'impose dans les formes symptomatiques et en cas de complication.

**Figure 1:**
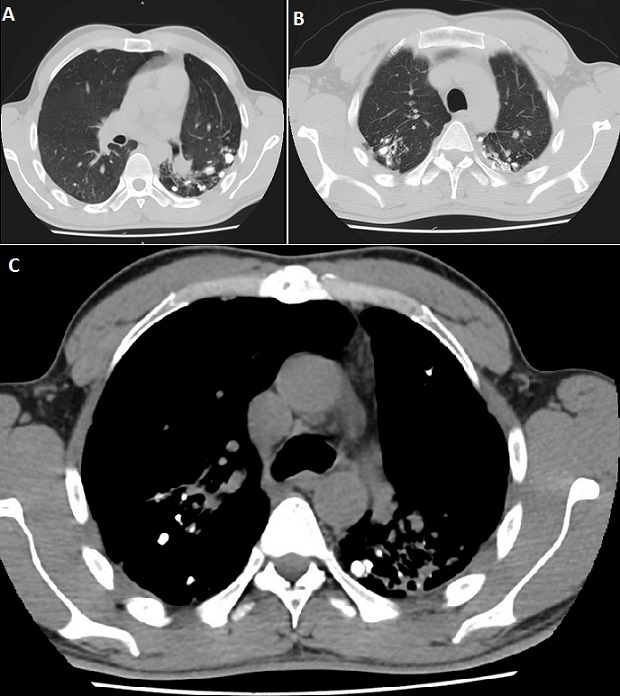
Scanner thoracique en fenêtre parenchymateuse montrant A) bronchectasies cylindriques et des calcifications parenchymateuses au niveau du lobe supérieur gauche, B) de multiples calcifications intrabronchiques des deux lobes supérieurs et C) scanner thoracique en fenêtre médiastinale montrant les calcifications parenchymateuses

